# Evaluation of the concentration of point defects in GaN

**DOI:** 10.1038/s41598-017-08570-1

**Published:** 2017-08-24

**Authors:** M. A. Reshchikov, A. Usikov, H. Helava, Yu. Makarov, V. Prozheeva, I. Makkonen, F. Tuomisto, J. H. Leach, K. Udwary

**Affiliations:** 1 Department of Physics, Virginia Commonwealth University, Richmond, VA 23284 USA; 2 Nitride Crystals, Inc, 181E Industry Ct., Ste. B, Deer Park, NY 11729 USA; 3 Saint-Petersburg National Research University of Information Technologies, Mechanics and Optics, 49 Kronverkskiy Ave, 197101 Saint Petersburg, Russia; 4 Department of Applied Physics, Aalto University, 00076 Aalto, Finland; 5 Kyma Technologies, Inc, Raleigh, North Carolina 27617 USA

## Abstract

Photoluminescence (PL) was used to estimate the concentration of point defects in GaN. The results are compared with data from positron annihilation spectroscopy (PAS), secondary ion mass spectrometry (SIMS), and deep level transient spectroscopy (DLTS). Defect-related PL intensity in undoped GaN grown by hydride vapor phase epitaxy increases linearly with the concentration of related defects only up to 10^16^ cm^−3^. At higher concentrations, the PL intensity associated with individual defects tends to saturate, and accordingly, does not directly correlate with the concentration of defects. For this reason, SIMS analysis, with relatively high detection limits, may not be helpful for classifying unidentified point defects in GaN. Additionally, we highlight challenges in correlating defects identified by PL with those by PAS and DLTS methods.

## Introduction

GaN is widely used in blue and white LEDs and lasers, as well as in other optical and electrical devices^1^. In order to achieve high performance and reliability of these devices, the concentrations of point defects unintentionally introduced during the material growth must be controlled. Point defects in semiconductors can be identified and their concentrations can be evaluated by several methods. Among these, capacitance methods such as capacitance-voltage (C-V) measurements and deep-level transient spectroscopy (DLTS)^2^ are routinely used for electrical characterization of semiconductors. A modification of the latter is optical DLTS (ODLTS)^3^, which allows in particular the detection and analysis of hole traps in *n*-type semiconductors. The capacitance methods require fabrication of a device such as a Schottky diode and can provide comprehensive information about deep-level defects. Positron annihilation spectroscopy (PAS) is efficient in detecting gallium vacancies (V_Ga_) and V_Ga_-containing complexes in GaN^4^. In particular, the concentration of negatively charged V_Ga_ determined from temperature-dependent PAS measurements was nearly equal to the total concentration of acceptors determined from temperature-dependent Hall effect measurements^5^. Secondary Ion Mass Spectrometry (SIMS) is helpful in revealing the chemical nature of impurities in semiconductors. However, the concentrations of impurities responsible for deep levels in undoped GaN are often below the detection limit. SIMS results may also be affected by contamination of the surface and by the presence of structural defects such as nanopipes or pits. Photoluminescence (PL) reveals radiative defects. It is especially useful for analysis of defects in wide-bandgap, direct-bandgap semiconductors, including GaN, which has a bandgap of 3.50 eV at low temperature^6, 7^.

All the above methods are able to determine the concentrations of at least some types of point defects in GaN. However, detailed analyses and quantitative comparisons of results obtained from the different methods are rare. In particular, based on conventional wisdom, the PL intensity is often assumed to be proportional to the concentration of related defects, whereas the validity of this assumption is not verified. Accordingly, attributions of PL bands to particular defects based on comparison of PL intensity with the concentration of defects obtained from other methods may be erroneous.

In this work, we use PL analysis to determine the concentration of several defect-related PL bands in GaN grown by hydride vapor phase epitaxy (HVPE). We have found that the PL intensity is proportional to the concentration of related defects up to only 10^16^ cm^−3^, near the detection limit of SIMS. The calculated concentrations of radiative defects in selected samples are compared with values obtained from other methods.

## Results

### Photoluminescence spectra from defects in undoped GaN

**Figure 1:**
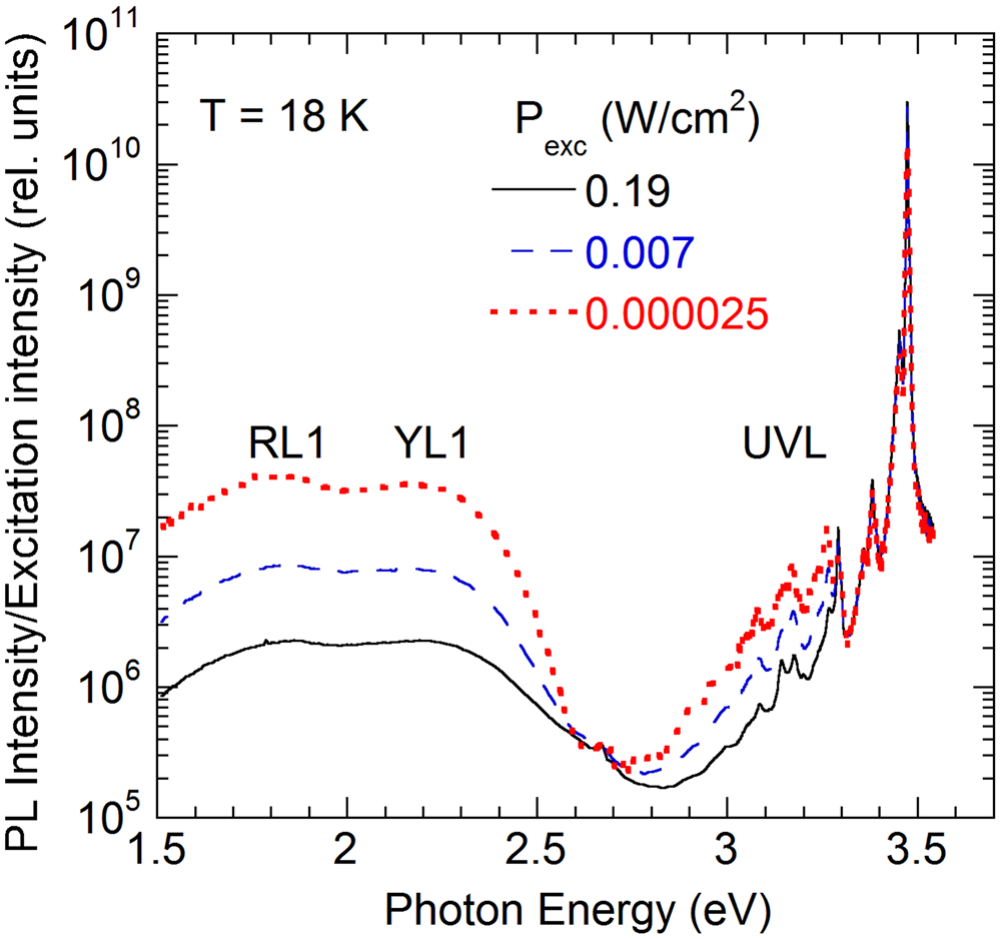
Low-temperature PL spectra from undoped GaN at selected excitation intensities. Sample H3. The PL intensity is divided by the excitation intensity for convenience of comparison.

Figure 1 shows low-temperature PL spectra from an undoped, 6.6 µm-thick GaN film grown on sapphire by HVPE. The exciton emission between 3.3 and 3.5 eV shows a sharp peak at 3.476 eV with the full-width at half maximum of 3.7 meV at 18 K. From the temperature dependence of PL, the peak at 3.476 eV is identified as an exciton bound to a neutral shallow donor. The exciton emission spectrum is typical of high-quality undoped GaN with a low concentration of defects^6^. At lower photon energies, relatively weak defect-related bands are observed: the ultraviolet luminescence (UVL) band with the zero-phonon line (ZPL) at 3.27 eV, the yellow luminescence (YL1) band with a maximum at 2.2 eV, and the red luminescence (RL1) band with a maximum at 1.8 eV. In some undoped GaN samples, the blue luminescence (BL1) band appears in the PL spectrum with the ZPL at 3.10 eV and maximum at 2.9 eV. This band is attributed to the Zn_Ga_ acceptor^8^. In sample H3, the BL1 band is very weak and can be detected only at elevated temperatures (at about 200 K), when the UVL band is quenched. The above nomenclature of defect-related PL bands in GaN was introduced in ref. 9.

**Figure 2:**
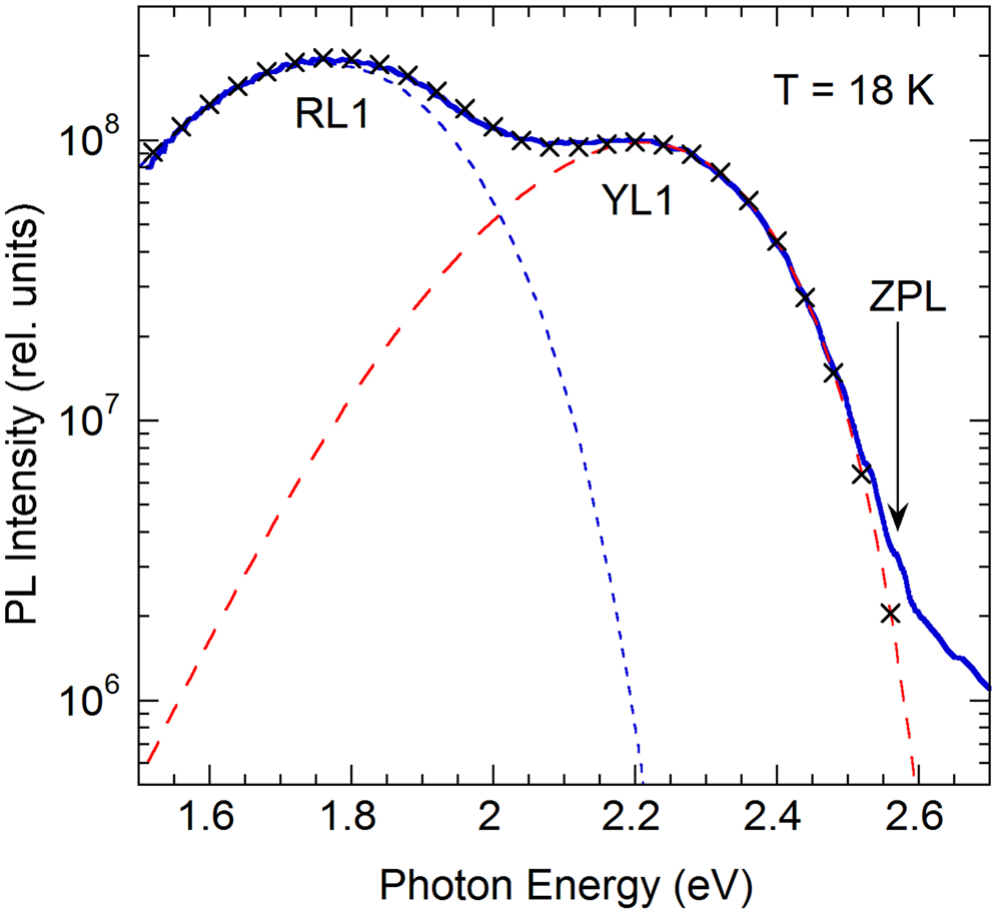
Part of the PL spectrum from undoped GaN. Sample H3. *T* = 18 K, *P*_*exc*_ = 5 × 10^−5^ W/cm^2^. The long-dashed and short-dashed lines show shapes of the YL1 and RL1 bands, respectively, obtained using Eq. (1) with the following parameters: ${I}_{\max }^{PL}=0.98\times {10}^{8}$, *S*_*e*_ = 7.4, *E*_0_^*^ = 2.67 eV, $\hslash {\omega }_{{\rm{\max }}}=2.21$ eV (for the YL1 band) and ${I}_{{\rm{\max }}}^{PL}=1.88\times {10}^{8}$, *S*_*e*_ = 10, *E*_0_^*^ = 2.33 eV, $\hslash {\omega }_{{\rm{\max }}}=1.76$ eV (for the RL1 band). The × symbols show the sum of two simulated bands. ZPL is the zero-phonon line of the YL1 band at 2.575 eV.

The defect-related PL bands can be better resolved and recognized at low excitation intensity. Figure 2 shows a part of the PL spectrum where the RL1 and YL1 bands are resolved using the following expression for the shape of the defect-related PL band obtained within a one-dimensional configuration-coordinate model^10^

1 $${I}^{PL}(\hslash \omega )={I}_{{\rm{\max }}}^{PL}\exp [-2{S}_{e}{(\sqrt{\frac{{E}_{0}^{\ast }-\hslash \omega }{{E}_{0}^{\ast }-\hslash {\omega }_{{\rm{\max }}}}}-1)}^{2}],$$

where ${I}_{\max }^{PL}$ is the intensity of a PL band at its maximum, *S*_*e*_ is the Huang-Rhys factor for the excited state (when the defect binds a hole), $\hslash \omega$ and $\hslash {\omega }_{{\rm{\max }}}$ are the photon energy and position of the band maximum, respectively; ${E}_{0}^{\ast }={E}_{0}+0.5\hslash \Omega$, *E*_0_ is the ZPL energy, and $\hslash \Omega$ is energy of the dominant phonon mode in the excited state. The parameters of the fit, given in the caption to Fig. 2, are very similar to the parameters of the RL1 and YL1 bands obtained in ref. 9. Moreover, careful analysis of the high-energy side of the YL1 and reveals its ZPL at 2.575 eV and three phonon replicas at multiples of 40 meV. This finding further confirms that the yellow band in this sample is caused by the same defect (the YL1 center) as in many other samples grown by different techniques^11^.

### Determination of the concentration of defects from photoluminescence

In this section, the effect of laser intensity on PL from GaN will be analyzed in detail to illustrate the method of finding the concentration of point defects from PL. The data for sample H3 will serve as an example here, with more examples given in refs 12–16. As will be shown below, this method requires information on the PL lifetime, *τ*_*i*_, and the absolute internal quantum efficiency (IQE) of PL, *η*_*i*_, for each PL band.

We will define the light extraction efficiency, *χ*, as the fraction of photons generated inside of a semiconductor that exits from the sample in direction of the collecting lens. Then, the integrated intensity of PL from a particular defect or recombination channel, ${I}_{i}^{PL}$, is

2 $${I}_{i}^{PL}=A\chi {\eta }_{i},$$

where *A* is a proportionality factor, which shows how efficiently the emitted light is detected by the PL setup. The parameter *A* is constant if the measurements are taken in identical conditions, and it is the same for different PL bands in a given sample. The parameter *χ* may vary from sample to sample (e.g., due to different smoothness of the surface), and it may have weak dependence on wavelength (which will be ignored here). For several GaN samples exhibiting unusually high radiative efficiency (called hereafter calibrated samples), the absolute IQE of PL was determined reliably with several independent methods^12, 13^. In particular, exceptionally high IQE (up to 96% at low temperature and low excitation intensity) was demonstrated by the observation of significant enhancement of different PL bands concurrently with thermal quenching of the BL1 band or with saturation of the BL1 band at high excitation intensity^12^. Assuming that the light extraction efficiency in different GaN samples is close to the extraction efficiency in these calibrated samples, *χ* ≈ *χ*_*cal*_, we can determine *η*_*i*_ for *i*-th PL band in a GaN sample from Eq. (2) by finding the ratios of the integrated PL intensities in this sample to the calibrated samples.

The intensity of the exciton emission increases linearly with the laser intensity, while the intensities of the defect-related PL bands increase more slowly, and their relative contribution to the PL spectrum decreases (Fig. 1). The absolute IQE of the exciton emission is about 5% at 18 K, while the total IQE of three defect-related PL bands does not exceed 2% in sample H3. With increasing temperature, the exciton emission is quenched due to dissociation of excitons, and its IQE decreases to 0.05% at 100 K. Intensities of the defect-related bands slightly increase (by a factor of ~1.5) in this temperature range.

Time-resolved PL measurements at 18 K reveal nonexponential decays of PL after the laser pulse for all three defect-related PL bands. Such behavior is common for transitions of electrons from shallow donors to deep defect levels, the so-called donor-acceptor pair (DAP) recombination. However, at 100 K, the decays of defect-related PL after the laser pulse become nearly exponential, so that the characteristic PL lifetime can be determined. The exponential decay of PL indicates that thermal emission of electrons from shallow donors to the conduction band becomes significant, and the DAP transitions are replaced with electron transitions from the conduction band to defect levels at 100 K. The PL lifetime of the UVL, YL1, and RL1 bands decreases with increasing temperature, in agreement with rising concentration of free electrons in this sample^9^. At 100 K, the PL lifetimes for the UVL, YL1 and RL1 bands in sample H3 are 15, 450, and 1000 µs, respectively. From these lifetimes, using electron-capture coefficients for different defects and their uncertainties^9^, we can find that $n=(2.1\pm 0.2)\times {10}^{16}$ cm^−3^ at 100 K.

**Figure 3:**
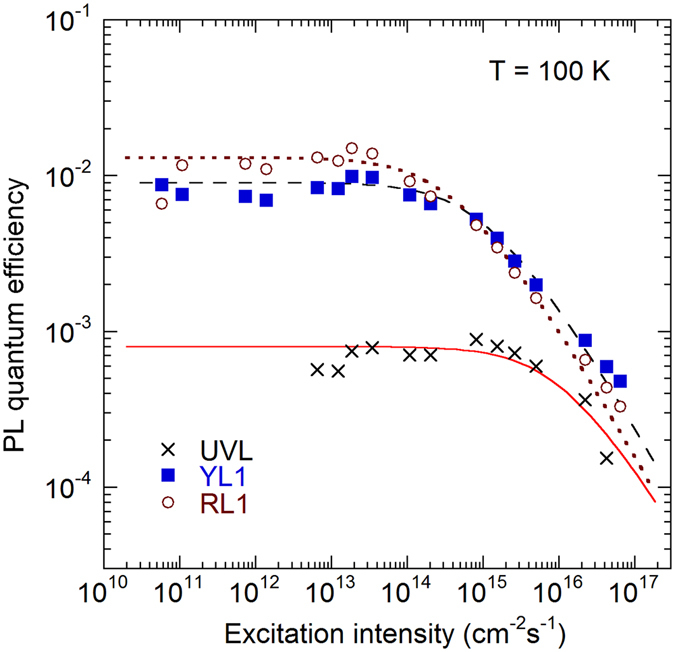
Dependence of PL quantum efficiency on the excitation intensity for PL bands in GaN. Sample H3 at 100 K. The lines are calculated using Eq. (3) with the following parameters: $\alpha =1.2\times {10}^{5}$ cm^−1^, *τ* = 15 µs, ${\eta }_{0}=8\times {10}^{-4}$, and $N=7\times {10}^{12}$ cm^−3^ for the UVL band, $\tau =450$ µs, ${\eta }_{0}=9\times {10}^{-3}$, and $N=2.4\times {10}^{14}$ cm^−3^ for the YL1 band, and *τ* = 1000 µs, ${\eta }_{0}=1.3\times {10}^{-2}$, and *N* = 2.4 × 10^14^ cm^−3^ for the RL1 band.

After finding PL lifetimes and the absolute IQE for defect-related PL bands, we can calculate the concentrations of related defects from analyses of the dependences of PL intensity on the excitation intensity, following the method described in refs 14–16. The dependences of the IQE of PL on excitation intensity for all three defect-related bands are shown in Fig. 3.

The IQE is nearly constant at very low excitation intensity (*η*_*i*_ = *η*_*i*0_), and it decreases at high excitation intensity. The decrease is attributed to the saturation of defects with photogenerated holes. It can be fit with the following formula^15, 16^

3 $${\eta }_{i}=\frac{{N}_{i}}{\alpha {\tau }_{i}{P}_{0}}\,\mathrm{ln}(1+\frac{\alpha {\tau }_{i}{\eta }_{i0}{P}_{0}}{{N}_{i}}),$$

where *η*_*i*0_ is the absolute IQE of the *i-*th PL band in the limit of low excitation intensities, *P*_0_ is the excitation intensity (the number of photons from the laser passing through a unit area of the sample surface per unit time), and *α* is the absorption coefficient for the incident photons (1.2 × 10^−5^ cm^−1^ at 325 nm)^17^. From the fit of the experimental data with Eq. (3), the concentrations of defects responsible for the UVL, YL1, and RL1 bands in GaN sample H3 are estimated as 7 × 10^12^, 2.4 × 10^14^, and 2.4 × 10^14^ cm^−3^, respectively. For another undoped GaN sample (H202), the concentrations of the same defects are 1 × 10^14^, 8 × 10^14^, and 2 × 10^15^ cm^−3^, respectively. Note that PL spectra from samples H3 and H202 are very similar.

The largest uncertainty in the determination of the concentration of defects from PL is caused by possible errors in the light extraction efficiency. From analysis of PL from several GaN samples, where the absolute IQE was high enough (that it could be estimated from the rise of PL bands caused by thermal quenching of the strongest PL band)^12, 13^, we determined that the $\chi /{\chi }_{cal}$ ratio may differ from unity by not more than half order of magnitude. Other sources of uncertainty are expected to cause much smaller errors.

*Relative* concentrations of radiative point defects can also be found from comparison of integrated PL intensities for different defects when the hole-capture coefficients for the defects are known. Indeed, it follows from rate equations for *n*-type GaN, accounting for Eq. (2), that^6^

4 $$\frac{{N}_{i}}{{N}_{j}}=\frac{{C}_{pj}}{{C}_{pi}}\frac{{I}_{i}^{PL}}{{I}_{j}^{PL}}$$

where *C*_*pi*_ and *C*_*pj*_ are the hole-capture coefficients for defects *i* and *j*. These coefficients can be found from analysis of the thermal quenching of PL^6, 14^.

Previously we have determined that *C*_*pi*_ ≈ 1 × 10^−6^ cm^3^/s for the UVL band (in the temperature range of 100–150 K) and *C*_*pi*_ ≈ 3 × 10^−7^ cm^3^/s for the YL1 band (in the temperature range of 450–700 K)^6, 18^. The thermal quenching of the RL1 band begins at very high temperature (above 500 K). From the quenching, we have estimated that *E*_*A*_ ≈ 1.0 eV and *C*_*pi*_ ≈ 1 × 10^−6^ cm^3^/s for the RL1 band. Note that the accuracy of these values is low (about ± 0.1 eV for *E*_*A*_ and half-order of magnitude for *C*_*pi*_), because the quenching with the largest slope was observed in the temperature range of 600–700 K, where the RL1 intensity decreased by only one order of magnitude. For the BL1 band, *C*_*pi*_ = 7 × 10^−7^ cm^3^/s in the temperature range of 200–300 K^8^.

Temperature dependence of *C*_*pi*_ for the studied defects is not known. First-principles calculations predict that it may rise with increasing temperature^19^. By using Eq. (4), we can find the ratios of the hole-capture coefficients at the *same temperature* for samples where the concentrations of defects are determined from the excitation intensity dependence. Since the parameters of the fit for sample H3 in Fig. 3 were obtained at 100 K, where *C*_*pi*_ = 1 × 10^−6^ cm^3^/s for the UVL band, we estimate from Eq. (4) that *C*_*pi*_ = 3.3 × 10^−7^ cm^3^/s for the YL1 band at this temperature; i.e., about the same as in the temperature range of 450–700 K. For the RL1 band, we estimate that *C*_*pi*_ = 4.7 × 10^−7^ cm^3^/s at 100 K. In a set of five undoped GaN samples grown by HVPE, for which we determined the concentration of defects from PL, the following mean values and the standard deviations of the mean were obtained for the hole-capture coefficients at *T* = 100 K: *C*_*pi*_ = (3.7 ± 1.6) × 10^−7^ cm^3^/s for the YL1 band, (2.9 ± 0.7) × 10^−7^ cm^3^/s for the RL1 band, and (4.9 ± 1.4) × 10^−7^ cm^3^/s for the BL1 band. We conclude that parameter *C*_*pi*_ for the RL1, YL1, and BL1 bands has a negligible temperature dependence, yet our estimates of the change in *C*_*pi*_ are rough. On the other hand, if the *C*_*pi*_(*T*) dependence was strong, we would see a significant increase of PL intensity in the temperature region before its thermal quenching, because the PL intensity in an *n*-type semiconductor is proportional to *C*_*pi*_ *N*_*i*_^6^. Such increase (which should be identical for different samples) is not observed experimentally. For samples with a very low intensity of defect-related PL bands, Eq. (4) is the only source for evaluation of the concentration of defects. In particular, for sample H3, the BL1 band is so weak that its dependence on excitation intensity cannot be studied. However, from Eq. (4), by taking intensities of the RL1, YL1, and BL1 bands at 200 K, we estimate that the concentration of the Zn_Ga_ defects responsible for the BL1 band in this sample is about 1 × 10^12^ cm^−3^, a value much lower than the detection limit of SIMS.

### Relation between the concentration of defects and photoluminescence intensity

**Figure 4:**
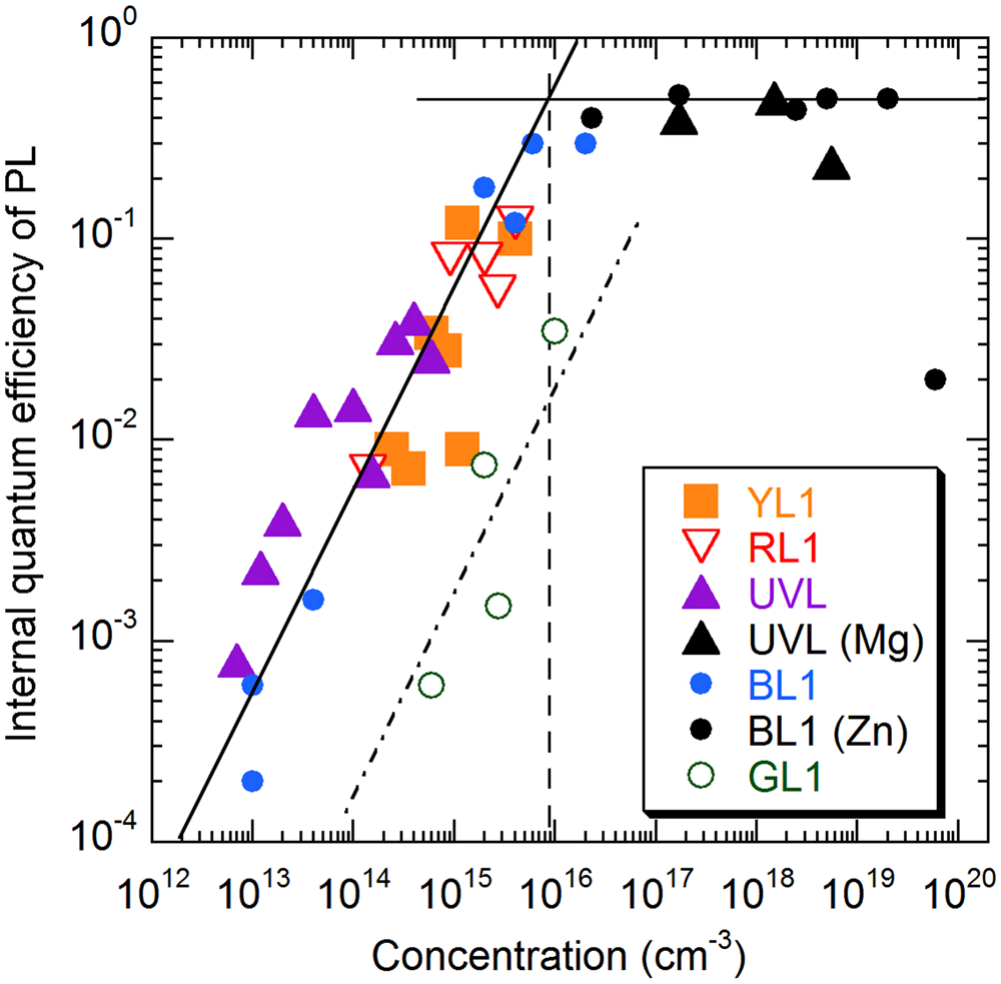
Dependence of PL quantum efficiency for defect-related PL bands in GaN on the concentration of related defects. The data are from ten undoped GaN samples, six Zn-doped GaN and three Mg-doped GaN samples, all grown by HVPE. The solid lines, which are guides for eye, show a linear dependence and a saturation at the value of ~0.5. The dashed vertical line indicates the concentration of defects above which the PL intensity from dominant defects has no linear relationship with the defect concentration. The data for the GL1 band (empty circles) follow linear dependence (dash-dotted line) but lie well below the data for the dominant PL bands (RL1, YL1, BL1, and UVL), because the related defect has much lower hole-capture cross-section.

By using the above-described approach, we have found a relation between the concentration of certain types of defects and associated PL intensities. Figure 4 includes the data for the dominant defect-related PL bands in conductive *n*-type GaN samples grown by HVPE. The IQE of PL in these data was determined by comparison with the calibrated samples, in the limit of low excitation intensities, where defects are not saturated with photogenerated holes. The concentration of defects was determined using Eq. (3), or from Eq. (4) when the intensity of a particular PL band was very weak. In addition to undoped GaN, where the concentrations of defects are low, we included the data for HVPE-grown GaN intentionally doped with Mg (the UVL band, black filled triangles) and doped with Zn (the BL1 band, black filled circles). The Mg-doped freestanding GaN samples with the thickness of about 0.5 mm were grown at Kyma Technologies, Inc. The Zn-doped GaN layers on sapphire substrates were grown at TDI, Inc. and studied in detail in ref. 13. The concentration of defects responsible for the UVL and BL1 bands in these samples is assumed to be equal to the concentrations of the Mg and Zn impurities, respectively, where the latter were determined with SIMS. All the samples were measured in identical conditions, and their integrated PL intensities were compared with the calibrated sample in order to find the IQE.

At low concentrations of defects (*N* < 10^16^ cm^−3^ for the RL1, YL1, BL1, and UVL bands), we observe a linear increase of PL intensity with the concentration of the related defects. At higher concentrations, the PL intensity is not rising because the IQE is close to unity for the dominant defect-related PL bands. The dependences for different defects of this group (RL1, YL1, BL1, and UVL) are similar because these defects have about the same hole-capture coefficient (between 3 × 10^−7^ and 10^−6^ cm^3^/s). These defects capture photogenerated holes very efficiently due to their large hole-capture cross-sections and cause strong PL even at moderate concentrations. For this reason, they dominate in the PL spectra of undoped GaN.

### Special case of the GL1 band

PL from defects with low hole-capture cross-sections may not be observed in the PL spectrum unless the concentration of these defects is high. An example is the green luminescence (GL1) band with a maximum at 2.40 eV in undoped GaN grown by HVPE. The GL1 band is rarely observed in the steady-state PL spectrum, because it is much weaker than the dominant PL bands (Fig. 1). However, it can be easily detected in time-resolved PL due to unusual properties of the GL1 defect^20^. This defect is a giant trap for electrons when it is positively charged. Such defects, being in a neutral charge state, are expected to capture holes less efficiently than negatively-charged acceptors. The dependence of the GL1 quantum efficiency on the concentration of the related defects is shown in Fig. 4 with empty circles. The dependence is nearly linear (the dash-dotted line), but the data points lie well below the dependence for the dominant defect-related PL bands. Our preliminary estimates for the hole-capture coefficient give a value of *C*_*pi*_ = (3.7 ± 1.3) × 10^−8^ cm^3^/s for the GL1 band using Eqs (3) and (4). The concentration of the GL1 defect was obtained from the excitation intensity dependences for only four HVPE GaN samples, and the value of *C*_*pi*_ may require refinement.

**Figure 5:**
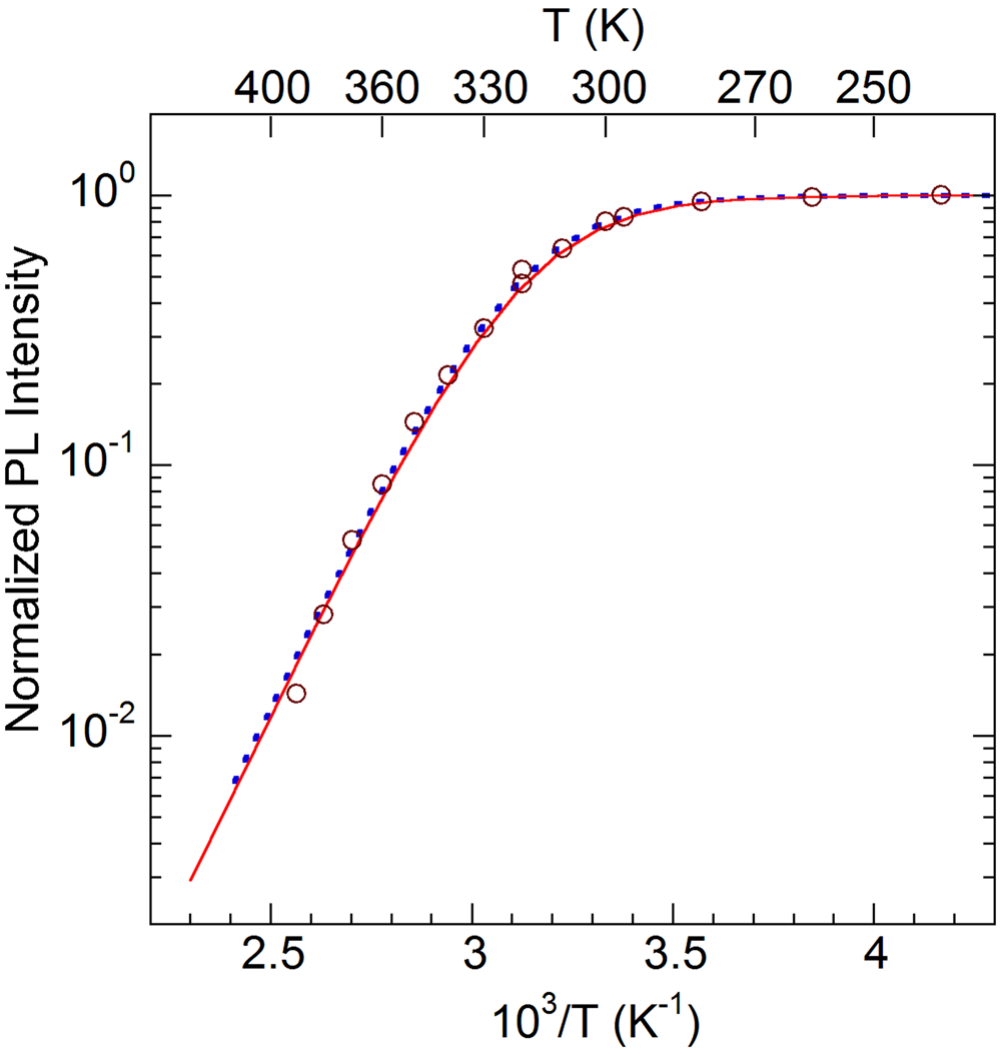
Temperature dependence of the GL1 band intensity. The lines are calculated using Eq. (5) with the following parameters: *τ*_0_ = 35 μs, *C*_*pi*_ = 1.5 × 10^−6^ cm^3^/s, and *E*_*A*_ = 0.55 eV (dotted line); ${\tau }_{0}=35\times {(T/280{\rm{K}})}^{3}$ μs, *C*_*pi*_ = 3 × 10^−8^ cm^3^/s, and *E*_*A*_ = 0.45 eV (solid line); *N*_*v*_ = 3.2 × 10^15^ *T* ^3/2^ cm^−3^ and *g* = 2 (both lines). The (1−*η*_0_) term in Eq. (5) is ignored due to low IQE.

Previously, we determined the hole-capture coefficient for the GL1 band as *C*_*pi* = _1 × 10^−6^ cm^3^/s from analysis of the thermal quenching of this band^21^. The temperature dependence of the PL intensity, *I* ^*PL*^, for the GL1 band normalized at *T* < 280 K is shown in Fig. 5. It can be fit with the following expression^14^:

5 $$\frac{{I}^{PL}(T)}{{I}_{0}}=\frac{1}{1+(1-{\eta }_{0}){\tau }_{0}{C}_{pi}{N}_{v}{g}^{-1}\exp (-{E}_{A}/kT)},$$

where *I*_0_, *η*_0_, and *τ*_0_ are the PL intensity, quantum efficiency, and PL lifetime, respectively, for the GL1 band before PL quenching begins (at about 280 K), *N*_*v*_ is the effective density of states in the valence band, *E*_*A*_ is the energy distance between the valence band maximum and the defect level, and *g* is its degeneracy. From the fit with constant *τ*_0_, shown with a dotted line in Fig. 5, we find that *E*_*A*_ = 0.55 eV and *C*_*pi*_ = 1.5 × 10^−6^ cm^3^/s. However, *τ*_0_ increases as ~*T* ^3^ between 100 and 280 K, which is explained using the model of an optically-generated giant trap^20^. If we account for the cubic temperature dependence of *τ*_0_ in Eq. (5) by extrapolating the low-temperature dependence into the region of thermal quenching (so that *τ*_0_ changes from 35 to 100 μs between 280 and 400 K), the best fit reveals the following parameters: *E*_*A*_ = 0.45 eV and *C*_*pi*_ = 3 × 10^−8^ cm^3^/s. Note that these are very rough estimates, because *τ*_0_ may increase slower than *T* ^3^. Nevertheless, the obtained *C*_*pi*_ agrees with the value of 3.7 × 10^−8^ cm^3^/s found at *T* = 100 K by using Eqs (3) and (4). The low value of the hole-capture coefficient may indicate that holes are captured by a neutral or even positively charged state.

The GL1 band was hitherto associated with the 0/+ transition level of the C_N_ defect^20, 21^. However, such attribution contradicts the fact that the GL1 band is strong in HVPE-grown GaN samples with very low concentration of carbon (10^15^–10^16^ cm^−3^), and it is not observed in MOCVD-grown GaN where the concentration of carbon is higher by one-two orders of magnitude. Identification of the GL1 defect requires further studies and is beyond the scope of the current work.

### Determination of the concentration of defects with other techniques

We have compared the concentrations of radiative defects in samples H3 and H202 found from PL with the concentrations of point defects obtained with other methods. SIMS data for these samples are given in Table 1. The total concentration of Si and O atoms, acting as shallow donors in GaN, is 0.93 × 10^17^ cm^−3^ (sample H3) and 1.21 × 10^17^ cm^−3^ (sample H202). These values agree with the concentration of free electrons determined from the temperature-dependent Hall effect measurements and time-resolved PL measurements (between 3.6 × 10^16^ and 7.5 × 10^16^ cm^−3^ at room temperature)^9^. The concentrations of carbon and chlorine are above the detection limit, whereas that of hydrogen appears to be below. Note that isolated hydrogen is expected to be a deep acceptor in *n*-type GaN^22, 23^, yet it may also form complexes with other defects^24^.

**Table 1:** Average concentration of impurities (in cm^−3^) from SIMS measurements in the region of 100–500 nm from the sample surface.

Impurity\Sample	H3	H202	Detection limit
C	4.6 × 10^16^	1.1 × 10^16^	2 × 10^15^
O	2.6 × 10^16^	3.2 × 10^16^	6 × 10^15^
Si	6.7 × 10^16^	8.9 × 10^16^	1 × 10^16^
H	6 × 10^17^	4 × 10^17^	(3–5) × 10^17^
Cl	3.9 × 10^16^	3.7 × 10^16^	1 × 10^15^

**Figure 6:**
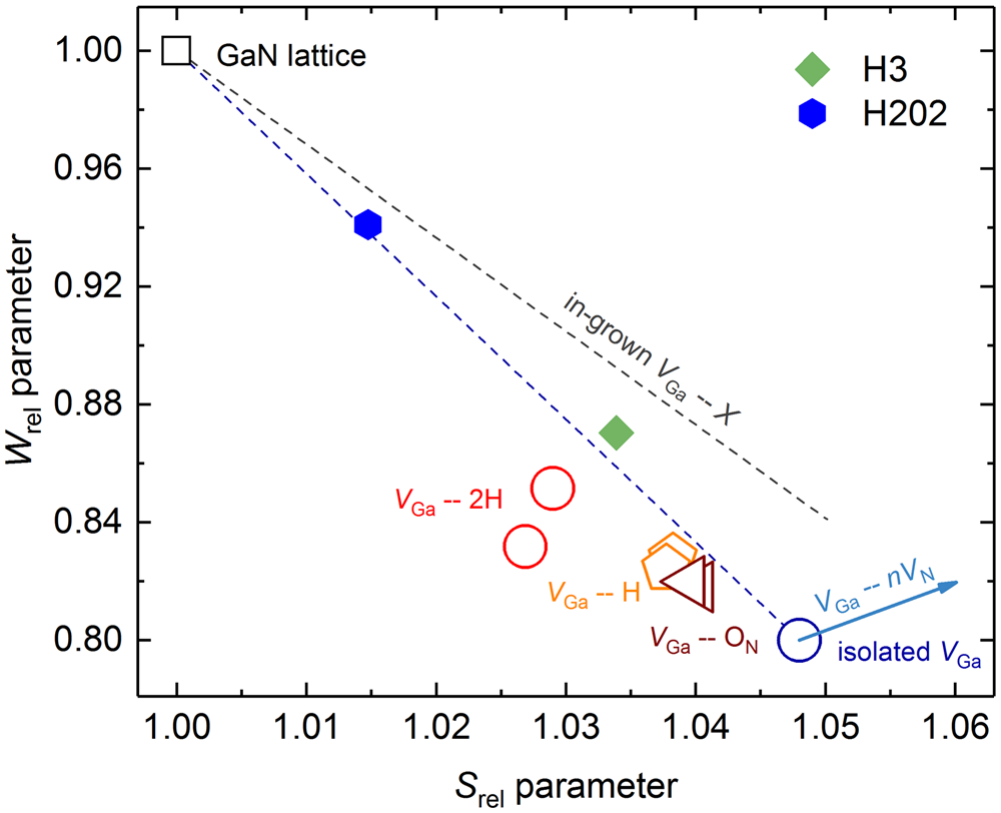
The *S* and *W* parameters for GaN. The layer-specific values for samples H3 and H202 are presented with diamond and hexagonal filled markers, respectively. Typical parameters for the GaN lattice, isolated V_Ga_, V_Ga_ − *m*H and V_Ga_O_N_ complexes are shown for comparison with empty symbols. The arrow indicates the signal evolution for V_Ga_ − *n*V_N_ with *n* ≥ 1. The upper dashed line shows the trend for in-grown V_Ga_ presumably complexed with other defects.

Gallium vacancy (V_Ga_)-related defects were studied with PAS in Doppler broadening mode at room temperature. Figure 6 shows the normalized *S* and *W* positron annihilation parameters for samples H3 and H202. The characteristic points correspond to isolated V_Ga_ (introduced by high-energy electron or ion irradiation)^25^ and to complexes of V_Ga_ with H and O_N_ according to our calculations. The blue arrow indicates the expected change in the (*S*, *W*) parameters for the signal from V_Ga_ complexed with nitrogen vacancies (V_Ga_ − *n*V_N_ with *n* ≥ 1)^26^. The upper dashed line presents the trend for the in-grown V_Ga_ supposedly complexed with other defects or impurities present in undoped GaN^5, 27, 28^. The data for two HVPE GaN samples lie close to the dashed line connecting the reference point “GaN lattice” obtained from defect-free GaN and the isolated V_Ga_. This suggests that the V_Ga_–related defects in these two samples are of the same type, possibly complexed with H, O and/or V_N_. It should be noted that the room temperature PAS experiments performed here do not provide any information on the charge state of the detected defects: they can be either neutral or negatively charged. Based on the (*S*, *W*) parameters of the lattice – defect line, the defect concentrations are estimated as 6 × 10^17^ cm^−3^ (sample H3) and 1 × 10^17^ cm^−3^ (sample H202). These values are expected to be correct on an absolute scale within a factor of 2–3, but relative error (between samples) should not exceed 10%.

The concentration of defects can also be determined with DLTS^2^. Hole traps in Schottky diodes prepared from the same piece (6.6 µm-thick GaN layer on 2-inch sapphire substrate) as sample H3 were studied in ref. 29 (labeled there “Improved HVPE”) by ODLTS using optical injection of holes with 5s-long optical (365 nm) pulses. The concentrations of the dominant hole traps with apparent activation energies of 0.7 eV and 0.9 eV were found to be 8.5 × 10^12^ and 6.3 × 10^12^ cm^−3^, respectively, in this sample. No other hole traps could be found with the ODLTS method. Additionally, from persistent photocapacitance, the total concentration of all hole traps was estimated in this work as 5 × 10^13^ cm^−3^. Polyakov *et al*. ^30^ also analyzed GaN samples (labeled “group 1”) similar to sample H202 studied in this work. In these samples, hole traps H3 (0.6 eV), H1 (0.9 eV), and H5 (1.1–1.2 eV) were observed with concentrations of 3 × 10^14^, 5 × 10^14^, and 5 × 10^14^ cm^−3^, respectively. These authors attributed the H1 and H5 traps to the V_Ga_Si_Ga_ and V_Ga_O_N_ complexes, respectively. Interestingly, the H5 trap was not observed in “Improved HVPE”, whereas PL spectra from these two samples are very similar. In addition to the hole traps, GaN samples studied by DLTS contained low concentrations of electron traps with energy levels closer to the conduction band. Note that in *n*-type GaN, the electron traps do not contribute to PL spectrum.

## Discussion

We have demonstrated that the concentration of defects contributing to the PL spectrum can be determined from analysis of PL (PL lifetime, PL IQE, and the dependence of PL intensity on excitation intensity). The results are reliable for *n*-type GaN, where the Fermi level is close to (or inside of) the conduction band. The total uncertainty in this method is about half-order of magnitude. This uncertainty arises mostly from possible errors in determination of the IQE due to different light extraction efficiencies in different GaN samples. The method can provide reliable data for the concentrations of defects in GaN down to 10^12^ cm^−3^. For lower concentrations, the PL bands are usually undetectable, because they are obstructed by PL bands from other types of defects with higher concentrations.

The dominant defect-related PL bands in GaN (RL1, YL1, BL1, and UVL) are caused by defects with very high hole-capture coefficients (3 × 10^−7^–10^−6^ cm^3^/s). For these defects, the PL intensity increases linearly with the concentration of defects up to about 10^16^ cm^−3^. At higher concentrations, the IQE is nearly constant (about 0.2–0.6 in the studied HVPE GaN samples). Then, for GaN intentionally doped with impurities or containing defects in as-grown material with concentrations exceeding 10^16^ cm^−3^, correlations between the PL intensity and the concentration of defects may be accidental and should be viewed with great caution. In particular, doping with acceptors with concentrations exceeding 10^17^ cm^−3^ may convert *n*-type into *p*-type or high-resistivity material, for which the PL intensity may be unusually high because of population inversion at low temperature^13^, and not because of high concentration of related defects. On the other hand, it may be abnormally low due to auto-compensation of acceptors with nonradiative donors with high concentration^31^, or due to high density of structural defects near the sample surface^32^.

Table 2 summarizes results from the SIMS, PAS, DLTS, and PL measurements for GaN samples H3 and H202. Several differences in the results obtained by different methods can be noticed. In particular, the positron annihilation measurements indicate high concentrations of V_Ga_-related defects (6 × 10^17^ cm^−3^ in sample H3). First principles calculations predict that a number of commonly expected V_Ga_-related defects are acceptors (V_Ga_, V_Ga_H_*i*_, V_Ga_O_N_, V_Ga_-2H_*i*_, and V_Ga_O_N_-H_*i*_)^24^. However, samples H3 and H202 contain low concentration of acceptors, according to the data obtained with other methods. Indeed, the Hall effect and time-resolved PL measurements conducted on sample H3 indicate that the room-temperature concentration of free electrons is (3–8) × 10^16^ cm^−3^, and the total concentration of impurities acting as shallow donors (Si and O) from SIMS measurements is about 1 × 10^17^ cm^−3^. Then, the total concentration of acceptors should be lower than ~5 × 10^16^ cm^−3^. This value agrees with our temperature-dependent Hall effect data. Relatively high concentrations of the V_Ga_-containing defects obtained by PAS measurements may indicate that the majority of these defects are ether electrically inactive or behave as deep donors in *n*-type GaN.

**Table 2:** Concentration of defects (in cm^−3^) from the SIMS, PAS, DLTS, and PL measurements.

Method	Defect	Sample H3	Sample H202
SIMS	C	4.6 × 10^16^	1.1 × 10^16^
	H	<6 × 10^17^	<4 × 10^17^
	Cl	3.9 × 10^16^	3.7 × 10^16^
	*N*_*D*_ (*D* = Si_Ga_ and O_N_)	9.3 × 10^16^	1.2 × 10^17^
PAS	V_Ga_-related defects	6 × 10^17^	1 × 10^17^
DLTS^29, 30^	H1 (0.9 eV)	6.3 × 10^12^	5 × 10^14^
	H3 (0.6 eV)		3 × 10^14^
	H4 (0.7–0.85 eV)	8.5 × 10^12^	
	H5 (1.1–1.2 eV)		5 × 10^14^
	Total of hole traps	5 × 10^13^	1.5 × 10^15^
	*N*_*D*_ − *N*_*A*_	(2–8) × 10^14^	1 × 10^17^
PL	RL1 (~1.2 eV)	2.4 × 10^14^	2 × 10^15^
	YL1 (0.916 eV)	2.4 × 10^14^	8 × 10^14^
	BL1 (0.40 eV)	1 × 10^12^	1 × 10^13^
	UVL (0.223 eV)	7 × 10^12^	1 × 10^14^
PL	Free electrons (*n*) at 300 K	3.6 × 10^16^	5.3 × 10^16^
Hall effect	Free electrons (*n*) at 300 K	7.5 × 10^16^	7 × 10^16^

The unusually low concentration of defects in sample H3 from capacitance measurements^29^ is not supported by the PL and Hall effect data. In particular, the concentration of uncompensated shallow donors is (2–8) × 10^14^ cm^−3^ according to C-V measurements, while the concentration of free electrons determined from the Hall effect and from time-resolved PL measurements is two orders of magnitude higher. This disagreement is probably the source of other apparent contradictions in the data for this sample. In particular, extremely low concentrations of traps calculated from ODLTS data for this sample directly follow from the very low concentration of uncompensated shallow donors obtained from C-V measurements. Indeed, the concentration of traps in DLTS (ODLTS) method is determined by multiplying the relative change in capacitance by the concentration of uncompensated shallow donors^2^. The total concentration of all hole traps found from persistent photocapacitance will also be scaled by the same factor, because the measured change in the built-in voltage is proportional to the ratio between the concentrations of hole traps and uncompensated shallow donors. Assuming that the concentration of uncompensated shallow donors in sample H3 is underestimated in C-V measurements by two orders of magnitude (to make it consistent with the SIMS and time-resolved PL data), we would obtain the concentration of the H1 trap in mid 10^14^ cm^−3^, close to the concentration of the YL1 defect.

It is very likely that the H1 trap and the YL1 center are the same carbon-related defect^7, 11, 33^. The concentrations of the H1 hole trap and the YL1 center in sample H202 are similar. It is necessary to mention that the concentration of the H1 trap in MOCVD-grown, undoped GaN is usually below 3 × 10^15^ cm^−3^ according to ODLTS measurements^34–36^, whereas the concentration of carbon from SIMS measurements is at least an order of magnitude higher in GaN samples grown by this technique. In our opinion, the H1 trap (YL1 center) is the only radiative defect reliably identified in GaN as a carbon-related defect (C_N_ or C_N_O_N_) with the transition level at 916 ± 3 meV above the valence band^11^. Other hole traps in undoped GaN grown by HVPE, namely the H2 (0.55 eV), H3 (0.65–0.7 eV), and H4 (0.85–0.9 eV)^38^, also known as HT2-HT4 traps^37^, are most probably nonradiative defects. The thermodynamic transition energy of the H5 trap (1.1–1.2 eV) agrees with that of the RL1 defect. However, the latter has relatively high concentration in sample H3, whereas the former was not observed in this sample (Table 2).

We would like to emphasize that the comparison of the concentrations of defects obtained from PL and from other methods (PAS and ODLTS) is preliminary. Obviously, larger sets of samples and closer interaction between experimentalists using different characterization methods are needed. The main purpose of the current work is to demonstrate the capabilities of the PL method. We can also foresee some limitations of the ODLTS technique when above-bandgap excitation is used (such as 365 nm light for GaN). Indeed, photogenerated holes are quickly (~10^–11^ s) swept by strong electric field (about 10^5^ V/cm) to the semiconductor surface (semiconductor-metal interface), where they may be trapped at surface states. Then, the change in capacitance after an optical injection pulse may be strongly affected by slow discharge of these states. Moreover, it appears from Fig. 2 in refs 34 and 38 that the ODLTS signal increases logarithmically with light intensity, and no saturation is achieved. Note that in original version of the ODLTS method, below-bandgap excitation is used, in which case free holes are not created and the capacitance change is attributed solely to filling of traps with holes^3^. The ODLTS measurements with below-bandgap excitation of GaN sometimes produce unreliable results, which can be attributed to the incomplete recharging of traps and nonuniform distribution of these traps in the space charge region^34^. The PL method is contactless and therefore free from above limitations.

In summary, we demonstrate that the concentration of radiative defects in GaN can be reliably determined from detailed PL measurements. At the same time, any apparent correlations between the PL intensity and the concentration of defects should be viewed with great caution when the latter exceeds 10^16^ cm^−3^, because they may be accidental. Other experimental techniques, such as PAS and ODLTS provide important complementary data because they can detect both radiative and nonradiative defects. The sources of differences between the concentrations of defects determined by using different experimental techniques should be investigated in detail in future studies. Importantly, conclusions about electrical or optical activity of certain kinds of defects should not be made based only on their concentrations. In particular, temperature-dependent PAS, allowing for direct observation of different charge states, should be employed.

## Methods

### Samples

We investigated in detail PL from 10 samples, 5–30 μm-thick, unintentionally doped GaN layers grown by HVPE on *c*-plane sapphire substrates. The concentration of free electrons in these samples is between 10^16^ and 10^17^ cm^−3^ at room temperature, as determined from the Hall effect and time-resolved PL measurements. Freestanding undoped and Mg-doped GaN samples were grown by HVPE at Kyma Technologies Inc. About 1–1.5 mm-thick GaN layers were separated from sapphire substrates and polished to remove surface damage. The concentration of Mg in Mg-doped samples was measured with SIMS.

### Experimental details

Steady-state PL was excited with an unfocused He-Cd laser (30 mW, 325 nm), dispersed by a 1200 rules/mm grating in a 0.3 m monochromator and detected by a cooled photomultiplier tube. Calibrated neutral-density filters were used to attenuate the excitation power density (*P*_exc_) over the range 10^−7^–0.2 W/cm^2^. Time-resolved PL was excited with a pulsed nitrogen laser (pulses with duration of 1 ns and repetition frequency of 6 Hz, and photon energy of 3.68 eV) and analyzed with an oscilloscope. A closed-cycle optical cryostat was used for temperatures between 15 and 320 K. The absolute internal quantum efficiency of PL, *η*, is defined as *η* = *I* ^*PL*^/*G*, where *I* ^*PL*^ is the integrated PL intensity from a particular PL band and *G* is the concentration of electron-hole pairs created by the laser per second in the same volume. To find *η* for a particular PL band, we compared its integrated intensity with the PL intensity obtained from a calibrated GaN sample^12, 13^. All of the samples were studied under identical conditions.

SIMS measurements have been carried out by the Evans Analytical Group. In analyses of undoped GaN samples (such as H3 and H202), a very low detection limit for carbon was achieved in vacuum by removing carbon adsorbed at the surface. The reduction in surface carbon resulted in less interference (and thus a lower background/detection limit) during the SIMS measurement of the underlying GaN region.

In PAS measurements, the trapping of positrons at negatively charged and neutral vacancy-type defects is observed as well-defined changes in the positron-electron annihilation radiation, e.g., as a narrowing of the momentum distribution of the annihilating positron-electron pair which is conserved in the annihilation event. The momentum distribution corresponds to the measured Doppler broadening of the annihilation photons. The shape of the Doppler broadened annihilation line is characterized with *S* and *W* parameters selected as fractions of positrons annihilating with low (|*p*_*L*_| < 0.4 a.u.) and high momentum (1.5 a.u. < |*p*_*L*_| < 3.9 a.u.) electrons, respectively^4^. The measurements were conducted at room temperature with an HPGe detector with a resolution of 1.25 keV at the 511 keV annihilation line with each spectrum containing approximately 1 × 10^6^ counts. The (*S*, *W*) parameters from the GaN layers were normalized to an MBE-grown Mg-doped GaN reference where no positron trapping into defects is observed.

The Doppler broadening of the annihilation line was modeled by using two-component density-functional theory (DFT) for electron-positron systems and related models. We used a 96-atom orthorhombic supercell to model the GaN lattice and Ga vacancy-related point defects. The VASP code^39, 40^, projector augmented-wave method^41, 42^, and the local-density approximation^43^ were used to describe the electronic structures. We use the limit of the two-component DFT, in which the localized positron does not affect the average electron density, and zero-component limits of the electron-positron correlation energy and enhancement factor. Due to cancellation and feedback effects, this scheme gives results that are consistent with more self-consistent modeling^44^. Ionic structures were relaxed taking into account the repulsive forces on ions due to the localized positron^45^. The Doppler broadening is modeled using the projector augmented-wave method^45, 46^ and the state-dependent enhancement scheme^47^. Prior to integrating the S/W parameters, the computational Doppler spectra were convoluted with the experimental resolution function.
